# Assessment of stressors and associated psychiatric co morbidities in migrant workers in a hilly state of India. A cross-sectional study

**DOI:** 10.1192/j.eurpsy.2026.10985

**Published:** 2026-07-31

**Authors:** S. Kaushik

**Affiliations:** Department of Health and Family welfare, Government of Himachal Pradesh, Baddi, District Solan, India

## Abstract

**Introduction:**

It has been observed that in the migrant workers psychosocial stressors affects mental health and are associated with psychiatric illness. As majority of the previous studies have analysed the subjects from general population only, the aim of our study is to explore the association between various stressors and mental health in the migrant workers.

**Objectives:**

This study was planned to identify various stressors and their role in individual’s mental health, in the migrant population working in industries and attending psychiatric outpatient department.

**Methods:**

The Study was conducted at the Civil Hospital, Baddi, Himachal Pradesh, India. Presumptive Stressful Life Events Scale (PSLES) — a stressful life events scale was used that is specifically designed for the Indian population. It was a cross-sectional observational study. The study was conducted from Jan 2024 to Dec 2024.

**Results:**

In total 500 patients,PSLES questionnaire was applied; majority of the patients were males and in the age group of 20-50 years. In our study, as per PSLES, the factors like loss of spouse, death of close family member, financial loss or problems (multiple loans), more number of kids, change of place or working conditions, marital conflict and excessive alcohol or drug use by family member were the common stressors identified in the studied population. The psychosocial stressors and there association with Psychiatric illness were analysed to look for their impact on migrant workers. The prevalence rate of nonspecific anxiety, panic disorder, adjustment disorder, and depressive illness were found to be 73.2%, 58.6%, 53.3% and 49.5%, respectively.

**Conclusions:**

In the present study, it was observed that even though some individuals may tolerate some stressors, but many are still very vulnerable to the psychiatric illness. The magnitude of manifestation depends primarily on the individuals and their social, cultural and economic differences.

**Key words:**

Migrant workers, stressors, psychiatric illness, and Presumptive Stressful Life Events Scale

**Disclosure of Interest:**

None Declared

